# Association Between Birth Spacing and Child Stunting in Indonesia: Evidence From a National Cross-Sectional Study

**DOI:** 10.34172/jrhs.13920

**Published:** 2026-08-15

**Authors:** Siti Masitoh, Dini Kurniawati, Bunga Astria Paramashanti, Yuni Purwatiningsih, Suparmi Suparmi, Nur Handayani Utami

**Affiliations:** ^1^Research Center for Public Health and Nutrition, the National Research and Innovation Agency, Cibinong Science Center, West Java, Indonesia; ^2^Health Systems Insight, Jakarta, Indonesia

**Keywords:** Birth intervals, Growth disorder, Family planning policy, Indonesia

## Abstract

**Introduction:**

Child stunting remains a major public health concern in Indonesia, driven by multifactorial determinants, including nutritional, socioeconomic, and reproductive factors. Although birth spacing has been hypothesized as an important determinant of child growth, national-level evidence remains limited. Thus, this study examined the association between birth spacing and the risk of stunting among children under five in Indonesia using nationally representative survey data.

**Study Design:**

A cross-sectional study.

**Methods:**

Data for this cross-sectional study were obtained from the 2024 Indonesia Nutrition Status Survey, which included 205,406 children aged 0–59 months. Stunting was defined as a height-for-age z-score<−2 SD based on WHO Child Growth Standards. In addition, birth spacings were categorized as<2 years and≥2 years. Complex sample multivariable logistic regression was applied to estimate adjusted odds ratios and 95% confidence intervals (CIs), controlling for child, maternal, and household characteristics.

**Results:**

Among 205,406 children, 18.4% were stunted. Moreover, children born less than two years after their preceding sibling had higher odds of stunting (1.12; 95% CI: 1.08–1.16; *P*<0.001) compared to those with longer birth spacing after adjusting for child, maternal, and household characteristics.

**Conclusion:**

Short birth spacing was significantly associated with child stunting in Indonesia, providing supportive evidence for considering birth spacing within broader stunting reduction and reproductive health strategies. Accordingly, future longitudinal studies are needed to clarify the causal pathways linking birth spacing and child nutritional outcomes.

## Introduction

 Child stunting remains one of the main public health challenges in low-income and middle-income countries. An estimated 23.2% of children under five were globally stunted in 2024, with the highest burden concentrated in Sub-Saharan Africa and South Asia.^1^ In Indonesia, although the prevalence of stunting has declined from 37.6% in 2013 to 19.8% in 2024, achieving the national target of 14.2% by 2029 remains a challenge.^2,3^ Stunting is associated with adverse consequences throughout the life course, including impaired cognitive development, lower educational attainment, reduced economic productivity, and an increased risk of poor health in adulthood.^4^ Given the long-term implications of stunting, identifying its modifiable determinants remains a priority for public health policies and interventions.

 The causes of stunting are multifactorial, extending beyond inadequate dietary intake and infectious disease exposure. Moreover, they encompass maternal health, economic conditions, sanitation, household environments, and broader structural determinants.^2,5,6^ Recognizing these challenges, the Indonesian government has prioritized stunting reduction as a national agenda through multisectoral efforts integrating nutrition-specific and nutrition-sensitive interventions.

 Among the various determinants of stunting, reproductive health factors, including birth spacing, warrant greater attention. It has important implications for both maternal and child health (MCH). Short birth spacing may deplete maternal nutrient reserves, limit recovery time from previous pregnancies, and reduce caregiving capacity, thereby negatively influencing child growth.^7,8^ According to global evidence, longer birth spacing (≥ 24 months) is significantly associated with a reduced risk of child undernutrition, including stunting.^9,10^ Although Indonesia has successfully reduced fertility rates through family planning (FP) programs, progress in maintaining optimal birth spacing has not advanced at the same pace, thus creating a knowledge gap regarding its association with child nutritional outcomes.

 It is noteworthy that birth spacing represents a potentially modifiable determinant that can be addressed through existing FP and maternal health services.^11,12^ Compared with socioeconomic and environmental factors, which frequently require broader structural changes and long-term investment ^13^, birth spacing can be influenced through established reproductive health policy interventions and programs.^11^ Furthermore, birth spacing occupies a strategic position at the intersection of reproductive health, maternal nutrition, and child health, making it particularly relevant for integrated approaches to stunting reduction.^14,15^ Accordingly, understanding its contribution may provide actionable evidence for strengthening existing policy interventions.

 Previous studies in Indonesia have largely emphasized socioeconomic, dietary, and environmental determinants of stunting (e.g., household income, parental education, sanitation access, and dietary diversity). While these factors remain crucial, there are a limited number of nationally representative analyses that have systematically examined the association between birth spacing and stunting, particularly using the most recent and comprehensive data.^16^ Additionally, the linkages between FP policies and MCH programs in relation to nutrition improvement strategies remain underexplored, despite their potential to accelerate the reduction in stunting. Therefore, this study aims to examine the association between birth spacing and stunting while providing evidence that may inform FP and maternal–child health strategies for stunting reduction in Indonesia.

## Methods

###  Study Design and Data Source

 This study considered a cross-sectional design using nationally representative data from the 2024 Indonesia Nutrition Status Study (Studi Status Gizi Indonesia: SSGI), conducted by the Ministry of Health in order to monitor nutritional indicators among children under five. In addition, the survey applied a multistage stratified sampling technique to ensure representativeness at the national and across all 38 provinces. Likewise, data were collected through structured interviews with mothers or primary caregivers and anthropometric measurements of children under five years.

###  Study Population and Sample

 The study population included all children aged 0–59 months recorded in the 2024 SSGI dataset. Further, the analytical sample was restricted to children with complete anthropometric measurements and complete data on relevant variables. Children with missing or implausible height-for-age (height-for-age z-score) data, as well as those from multiple births, were excluded from the analysis. Finally, a total of 205,406 children met these criteria and were included in the study.

###  Variables

 The primary outcome variable was stunting among children aged 0–59 months, which was defined according to the World Health Organization (WHO) Child Growth Standards. Additionally, a child was classified as stunted if his or her height-for-age z-score was less than −2 standard deviations (SD) from the WHO median reference. The key independent variable was the birth spacing, defined as the time (in years) between the index child’s birth and the preceding sibling’s birth. Similarly, birth spacing was characterized as the interval (in years) between the index child’s birth and the preceding sibling’s birth. Birth spacing was classified as < 2 years and ≥ 2 years based on the WHO recommendation that women should wait at least 24 months after a live birth before attempting the next pregnancy to reduce the risk of adverse maternal, perinatal, and infant outcomes.^17^ Considering the predefined birth-spacing categories available in the SSGI dataset, the same classification was applied in this study in order to ensure consistency with the original data. To account for potential confounding, several covariates were also included in the multivariable models based on prior literature and biological plausibility. These covariates were grouped into three domains. Child characteristics included age (0–11, 12–23, 24–35, 36–47, and 48–59 months) and gender (male or female). Moreover, maternal traits comprised educational attainment (no education/primary, secondary, or higher) and employment status (unemployed or employed). Ultimately, household factors included household wealth quintile (poorest, poorer, middle, richer, or richest) and place of residence (urban or rural).

###  Statistical Analysis

 All analyses accounted for the multistage sampling design and survey weights of the 2024 Indonesia Nutrition Status Survey (SSGI 2024) to ensure national representativeness. First, descriptive statistics were calculated to summarize sample characteristics. In addition, categorical variables were presented as weighted proportions with 95% confidence intervals (CIs), stratified by stunting status. Differences between groups were assessed using the chi-square test.

 Furthermore, multivariable logistic regression analysis was conducted to examine the association between birth spacing and stunting. Birth spacing was the main independent variable. All theoretically relevant variables and those with a *P*-value < 0.25 in bivariate analysis were entered into the initial multivariable model. Further, this threshold was selected in accordance with the purposeful selection approach, which advocates a more inclusive screening criterion to ensure that potentially important confounding variables are not prematurely excluded from multivariable analyses.^18^ Moreover, confounding was evaluated by sequentially removing non-significant variables (*P* ≥ 0.05) and comparing adjusted with crude odds ratios; a change > 10% indicated confounding. The final model retained significant variables (*P* < 0.05) and identified confounders. Additionally, adjusted odds ratios (aORs) with corresponding 95% CIs were reported to quantify the strength of associations. All analyses were performed using SPSS, version 20 (IBM Corporation, Armonk, NY, USA).

## Results

 A total of sample 205,406 children aged 0–59 months were included in the analysis. Table 1 summarizes the sample characteristics by stunting status. Overall, 18.4% of children were stunted, which is consistent with the national prevalence reported in SSGI 2024. Likewise, the prevalence of stunting varied significantly across most sociodemographic characteristics, except for birth spacing. Children born after a short birth spacing (< 2 years) had a similar likelihood of being stunted compared to those with a birth spacing of ≥ 2 years (18.6% vs. 18.4%; *P*= 0.651). The crude OR (cOR = 1.01; 95% CI: 0.96–1.07) represented no significant association between birth spacing and stunting. Birth spacing was treated as the main independent variable in the analysis. It should be noted that the association between birth spacing and stunting may be influenced by other factors. Hence, even though the crude association appeared nonsignificant, the multivariable analysis was required to assess the association between birth spacing and stunting while accounting for potential confounders.

**Table 1:** Sample Characteristics of Children Under Five by Stunting Status (SSGI 2024)

**Characteristics**	**Stunted**		**Not-Stunted**		***P*** **-Value**	**Crude OR (95% CI)**
	**Number**	**%**	**Number**	**%**		
Birth spacing (year)						
≥ 2 < 2	36282	18.4	144992	81.6		Ref.
	5083	18.6	19049	81.4	0.651	1.01 (0.96, 1.07)
Age (months)						
0–11 12–23 24–35 36–47 48–59	2317	6.6	28682	93.4		Ref.
	8608	18.8	32855	81.2	0.001	3.29 (3.04, 3.57)
	10863	22.7	33616	77.3	0.001	4.18 (3.86, 4.52)
	10583	21.6	34657	78.4	0.001	3.92 (3.62, 4.24)
	8994	19.2	34231	80.8	0.001	3.39 (3.13, 3.67)
Gender						
Female Male	18995	17.5	80894	82.5		Ref.
	22370	19.3	83147	80.7	0.001	1.12 (1.09, 1.16)
Education level						
Higher Secondary No education/primary	4274	12.9	26201	87.1		Ref.
	24141	17.7	99634	82.3	0.001	1.44 (1.36, 1.53)
	12950	23.2	38206	76.8	0.001	2.03 (1.90, 2.16)
Employment status						
Employed Unemployed	14392	17.7	59856	82.3		Ref.
	26973	18.8	104185	81.2	0.001	1.07 (1.04, 1.11)
Household wealth quintile						
Richest Richer Middle Poorer Poorest	5197	11.9	35946	88.1		Ref.
	7227	16.2	34704	83.8	0.001	1.44 (1.35, 1.53)
	7752	17.7	32581	82.3	0.001	1.59 (1.50, 1.70)
	9318	20.7	32333	79.3	0.001	1.94 (1.83, 2.06)
	11871	27.6	28477	72.4	0.001	2.83 (2.66, 3.00)
Place of residence						
Urban Rural	18912	16.7	88851	83.3		Ref.
	22453	21.0	75190	79.0	0.001	1.33 (1.29, 1.38)
	Total	41365	18.4	164041	81.6		

*Note*. OR: Odds ratio: CI: Confidence interval; Ref.: Reference;SSGI 2024: The 2024 Indonesia Nutrition Status Survey.

 Among child characteristics, age showed the strongest and most consistent association with stunting. Compared to infants aged 0–11 months, the odds of being stunted sharply increased among those aged 12–23 months (OR = 3.29; 95% CI: 3.04–3.57) and peaked in the 24–35 months group (OR = 4.18; 95% CI: 3.86–4.52). Although the risk slightly declined in older children, it remained markedly higher than in infancy, reflecting the critical period during the transition from exclusive breastfeeding to complementary feeding. Similarly, male children were more likely to be stunted than females (19.3% vs. 17.5%; *P* < 0.001), with 12% higher odds (OR = 1.12; 95% CI: 1.09–1.16).

 Regarding maternal factors, educational attainment was a strong predictor of child nutritional status. Compared with mothers holding higher education, those with secondary education had 1.44 times higher odds of having a stunted child (OR = 1.44; 95% CI: 1.36–1.53). However, children of mothers with no or only primary education were twice as likely to be stunted (OR = 2.03; 95% CI: 1.90–2.16). Likewise, employment status demonstrated a modest yet significant effect; more precisely, children of unemployed mothers were slightly more likely to be stunted than those of employed mothers (OR = 1.07; 95% CI: 1.04–1.11).

 Finally, household factors displayed a clear socioeconomic gradient. Children from poorer and poorest quintiles, compared with those from the richest households, had substantially higher odds of stunting, reaching nearly threefold in the poorest group (OR = 2.83; 95% CI: 2.66–3.00; *P* < 0.001). In addition, stunting was more prevalent among rural children (21.0%) in comparison with their urban counterparts (16.7%), with 33% higher odds (OR = 1.33; 95% CI: 1.29–1.38).

Table 2 presents the results of the multivariable logistic regression examining the association between birth spacing and stunting after adjusting for child, maternal, and household characteristics. The adjusted analysis revealed that birth spacing remained a significant predictor of stunting. Based on the results children born after a short birth spacing (< 2 years) had 1.12 times higher odds of having a stunted child compared to those born after a longer spacing (aOR = 1.12; 95% CI: 1.08 –1.16; *P* < 0.001). In general, our findings confirmed that short birth spacing is associated with higher odds of stunting, even after accounting for child, maternal, and household features.

**Table 2:** Multivariable Logistic Regression of Birth Spacing and Stunting Among Children Under Five (SSGI 2024)

**Characteristics**	**Coefficient B**	**Robust SE**	***P*** **-Value**	**Adjusted OR (95% CI)**
Birth spacing (year)				
≥ 2 < 2				Ref.
	0.112	0.017	0.001	1.12 (1.08, 1.16)
Age (months)				
0–11 12–23 24–35 36–47 48–59				Ref.
	1.193	0.025	0.001	3.30 (3.14, 3.46)
	1.399	0.024	0.001	4.05 (3.86, 4.25)
	1.348	0.024	0.001	3.85 (3.67, 4.04)
	1.193	0.025	0.001	3.30 (3.14, 3.46)
Gender				
Female Male				Ref.
	0.143	0.011	0.001	1.15 (1.13, 1.18)
Education level				
Higher Secondary No education/primary				Ref.
	0.124	0.020	0.001	1.13 (1.09, 1.18)
	0.282	0.022	0.001	1.33 (1.27, 1.39)
Employment status				
Employed Unemployed				Ref.
	0.039	0.012	0.001	1.04 (1.02, 1.07)
Household wealth quintile				
Richest Richer Middle Poorer Poorest				Ref.
	0.295	0.021	0.001	1.34 (1.29, 1.40)
	0.397	0.021	0.001	1.49 (1.43, 1.55)
	0.577	0.021	0.001	1.78 (1.71, 1.85)
	0.900	0.021	0.001	2.46 (2.36, 2.56)
Place of residence				
Urban Rural				Ref.
	0.140	0.012	0.001	1.15 (1.12, 1.18)

*Note*. OR: Odds ratio: CI: Confidence interval; Ref.: Reference;SSGI 2024: The 2024 Indonesia Nutrition Status Survey; SE: Standard error.

## Discussion

 The findings of our study demonstrated that children born after a short birth spacing were significantly more likely to experience stunting compared with those born after an optimal spacing, even after adjusting for socioeconomic, child, and maternal factors. While no significant association was observed in the crude analysis, the association emerged after multivariable adjustment, suggesting that the unadjusted relationship was influenced by confounding factors. Although the magnitude of the relationship was modest (aOR = 1.12; 95% CI: 1.08–1.16), the finding is important from a population health perspective. In addition, multiple regression analyses indicated that child characteristics (age and gender) were remarkably associated with stunting. Among maternal factors, education level and employment status showed significant effects, while socioeconomic status and type of residence were also related to stunting. Given Indonesia’s large birth cohort, a 12% higher risk among children born after short birth spacing represents a considerable number of affected children nationwide. Furthermore, the modest effect size is expected, as stunting is a multifactorial condition that is influenced by nutritional, socioeconomic, and maternal health factors. Nevertheless, the consistent and statistically significant association suggests that short birth spacing may display a potentially modifiable reproductive factor that warrants consideration within FP and maternal-child health programs.

 Some previous studies conducted in low-income and middle-income countries consistently reported that birth spacing with interval greater than 24 months was associated with a lower risk of child stunting and underweight.^19,20^ While other studies revealed that controlling the birth interval of 36–48 months yielded the strongest protective effect on the likelihood of stunting, wasting, and undernutrition among children under five when compared to less than 24 months.^9,12^ These studies indicate that both biological and behavioral mechanisms underpin the relationship.

 Biologically, short intervals may not allow adequate time for maternal nutritional recovery after pregnancy and lactation; this phenomenon is frequently described as “maternal depletion syndrome,” where essential micronutrients (e.g., iron, folate, calcium, and protein) are not adequately replenished between pregnancies.^21,22^ Some studies have reported that short birth spacing is linked with adverse birth outcomes, including low birth weight, preterm birth, and intrauterine growth restriction, which are recognized predictors of stunting.^23,24^ Based on these findings, insufficient maternal recovery between pregnancies compromises maternal health and fetal growth, perpetuating the cycle of malnutrition.

 Beyond biological factors, social and behavioral mechanisms further contribute to the observed relationship. For instance, closely spaced births intensify competition for household resources, particularly in low-income families, leading to reduced parental attention, shorter breastfeeding duration, and inadequate complementary feeding.^25,26,27^ Previous evidence highlights that sibling competition and limited caregiving capacity contribute to lower dietary diversity and persistent undernutrition. Therefore, adequate intervals between pregnancies may contribute to improved maternal nutritional recovery and child growth outcomes.

 From a policy perspective, these findings can be considered policy-relevant evidence for Indonesia’s ongoing efforts to reduce stunting through a life-course and reproductive health lens. Although the national FP program has effectively reduced fertility rates, awareness of the health benefits of optimal birth spacing remains limited.^28^ Nonetheless, the observed association between birth spacing and stunting provides supportive evidence for considering birth spacing within broader stunting reduction strategies, as has been emphasized in several Asia-Pacific countries.^29^ Additionally, the existing FP campaigns and communication initiatives may consider incorporating messages on optimal birth spacing alongside family size preferences. In this context, collaboration between the Ministry of Health and National Population and FP Board (BKKBN) may further support the integration of reproductive health and nutrition objectives within existing programs.

 Programmatically, the observed association between birth spacing and stunting provides supportive evidence for taking into consideration the postpartum FP (PPFP) services within broader MCH strategies.^30^ PPFP may represent a relevant component of continuum of maternal and child care, including access to modern contraceptives immediately after childbirth, counselling during antenatal, postnatal, and child immunization visits. Likewise, the existing MCH, nutrition, and FP programs may offer opportunities to incorporate messages on optimal birth spacing. In addition, routine monitoring of PPFP indicators and the availability of contraceptive services at primary health care facilities may be regarded as potential areas for program strengthening. While the present study could not establish causal pathways, these findings suggest that birth spacing may be an important factor to consider within integrated reproductive, maternal, and child health programs aimed at improving child health outcomes.

 Our findings provided supportive evidence for the importance of PPFP within MCH programs. The integration of PPFP indicators into routine MCH reporting may facilitate performance at the facility level.^31,32^ Moreover, ensuring the availability of adequate contraceptive supplies, trained midwives, and decision-support tools at Community Health Centers (Puskesmas) and Integrated Health Posts (Posyandu) can be considered part of broader efforts to support informed postpartum contraceptive choices.^33,34^ Stunting prevention is inherently multisectoral, requiring coherence across reproductive health, maternal nutrition, and early childhood programs.^35^ Thus, policy alignment among Ministry of Health, the National Population and FP Board, and local governments may support better synchronization of planning, budgeting, and monitoring.

 A key strength of this study was the use of nationally representative survey data from the most recent Indonesia Nutrition Status Survey (SSGI 2024). Further, the large sample size and application of multivariable logistic regression allowed for adequate adjustment of essential confounders at the child, maternal, and household levels, thereby increasing the internal validity of our findings. Ultimately, the inclusion of stratified sampling weights ensured national and provincial representativeness, enhancing generalizability.

 However, several limitations should be acknowledged. First, the cross-sectional design prevented causal inference and limited the ability to determine temporal relationships between exposure and outcome. Moreover, birth spacing relied on maternal recall, which may be subject to recall bias. Additionally, the dataset lacked information on FP support (e.g., contraceptive use, postpartum counselling, and contraceptive discontinuation) as well as several potentially important confounders, including maternal age, parity, pregnancy intention status, maternal nutritional status, and breastfeeding practices, which may have resulted in residual confounding. Eventually, the study did not assess paternal factors or cultural norms that may influence birth spacing decisions, which may have introduced unmeasured sociocultural bias. Accordingly, future research using longitudinal or prospective cohort data is needed to better understand the temporal relationship between birth spacing and child growth outcomes and further explore the mechanisms underlying the observed association. Finally, it is recommended that future mixed-method studies explore sociocultural norms, parental decision-making processes, and caregiving practices related to birth spacing across diverse Indonesian settings.

**Highlights**

- Birth spacing < 2 years increased the odds of stunting by 12% after adjustment.
- Child characteristics, maternal education, employment, socioeconomic status, and residence were associated with stunting.
- Our findings confirmed the inclusion of birth spacing in stunting prevention programs.

## Conclusion

 This study revealed that short birth spacing was associated with higher odds of child stunting in Indonesia. Our findings can be considered policy-relevant evidence supporting the inclusion of birth spacing within broader stunting reduction and reproductive health initiatives. Although causal relationships could not be established due to the cross-sectional design, future longitudinal studies are required to gain a better understanding of the pathways linking birth spacing and child nutritional outcomes.

## Acknowledgments

 We gratefully acknowledge the Ministry of Health of the Republic of Indonesia for granting access to the data utilized in this study.

## Artificial Intelligence Use Disclosure

 The authorssolely used ChatGPT (OpenAI) to assist with editing the language and improving the clarity of the manuscript. Nonetheless, the tool was not employed for study design, data collection, data analysis, interpretation of the findings, or the development of scientific conclusions. Further, the authors reviewed, revised, and approved all AI-assisted content. Ultimately, they take full responsibility for the accuracy and integrity of the final manuscript.

## Competing Interests

 The authors have no competing interests associated with the material presented in this study.

## Ethical Approval

 The 2024 Indonesian Nutrition Status Survey (SSGI) received ethical approval from the Health Research Ethics Committee of Poltekkes Jakarta II (approval No. DP.04.03/I/KE/230/2024). In addition, written informed consent was obtained from all participants before data collection. This secondary analysis of anonymized public-use data required no additional ethical approval.

## Funding

 None.
